# 39. Anti-Spike Monoclonal Antibody Therapy for Kidney Transplant Recipients with COVID-19

**DOI:** 10.1093/ofid/ofab466.039

**Published:** 2021-12-04

**Authors:** Anna Hardesty, Elizabeth Klein, Kendra Vieira, Dimitrios Farmakiotis

**Affiliations:** 1 Brown University Internal Medicine Residency, Providence, RI; 2 Warren Alpert Medical School of Brown University, Providence, Rhode Island; 3 Divisions of Infectious Diseases,the Warren Alpert Medical School of Brown University, providence, Rhode Island; 4 Division of Infectious Diseases, The Warren Alpert Medical School of Brown University, providence, Rhode Island

## Abstract

**Background:**

Organ transplant recipients may not mount an adequate immune response to COVID-19 infection, and therefore may benefit greatly from passive immunization with anti-spike monoclonal antibodies (mAb), which have been shown to decrease hospitalization rates in the general outpatient population. We evaluated the efficacy of mAb therapy in decreasing hospitalizations or emergency room (ER) visits among kidney transplant recipients (KTR) with COVID-19.

**Methods:**

We identified KTR with COVID-19 between 3/1/2020 and 4/30/2021. Patients were excluded if they had multiorgan transplant or hospital-acquired COVID-19. Data were analyzed by Cox regression with mAb administration as time-dependent variable, and the day of symptom onset as baseline.

**Results:**

We studied 95 KTR; 20 received mAb. Comorbidities and immunosuppression were balanced between the two groups. mAb administration was associated with a significant decrease in hospitalizations or ER visits (15 vs. 76%, P< 0.001). This association remained significant after adjustment for confounders and by analyzing mAb administration as a time-dependent variable (Table: adj. HR 0.2, P=0.04). No KTR who received mAb died or required mechanical ventilation. Black or Hispanic KTR were less likely to receive mAb and more likely to be admitted to the hospital or visit the ER (Table).

Table

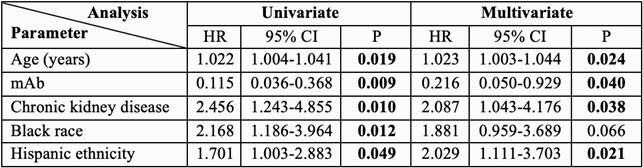

Factors significantly associated with hospitalization or ER visit.

**Conclusion:**

In our KTR population, mAb therapy for COVID-19 may have helped decrease hospitalizations and ER visits. Healthcare inequities, including access to investigational treatments, were exacerbated by the COVID-19 pandemic. Acknowledging the nonconcurrent control group as a limitation, we found a strong signal for benefit from mAb treatment. Antiviral mAb are a promising therapeutic modality for immunosuppressed patients.

**Disclosures:**

**Dimitrios Farmakiotis, M.D.**, **Astellas** (Grant/Research Support)**Merck** (Grant/Research Support)**Viracor** (Grant/Research Support)

